# Gamma oscillations modulate working memory recall precision

**DOI:** 10.1007/s00221-021-06051-6

**Published:** 2021-07-05

**Authors:** Lyall Thompson, Janine Khuc, Maria Silvia Saccani, Nahid Zokaei, Marinella Cappelletti

**Affiliations:** 1 Department of Psychology, Goldsmiths, University of London, Lewisham Way, London, SE14 6NW UK; 2 Institute of Cognitive Neuroscience, University College London, 17 Queen Square, London, WC1N 3AR UK; 3 Department of Experimental Psychology, South Parks Road, Oxford, OX1 3UD UK; 4 Department of Psychiatry, Oxford Centre for Human Brain Activity, Wellcome Centre for Integrative Neuroimaging, University of Oxford, Oxford, OX3 7JX UK

**Keywords:** Working memory, Attention, Inhibition, Gamma oscillations, tACS

## Abstract

Working memory (WM)—the ability to keep information in mind for short periods of time—is linked to attention and inhibitory abilities, i.e., the capacity to ignore task-irrelevant information. These abilities have been associated with brain oscillations, especially parietal gamma and alpha bands, but it is yet unknown whether these oscillations also modulate attention and inhibitory abilities. To test this, we compared parietal gamma-transcranial alternating current stimulation (tACS) to alpha-tACS and to a non-stimulation condition (Sham) in 51 young participants. Stimulation was coupled with a WM task probing memory-based attention and inhibitory abilities by means of probabilistic retrospective cues, including informative (valid), uninformative (invalid) and neutral. Our results show that relative to alpha and sham stimulation, parietal gamma-tACS significantly increased working memory recall precision. Additional post hoc analyses also revealed strong individual variability before and following stimulation; low-baseline performers showed no significant changes in performance following both gamma and alpha-tACS relative to sham. In contrast, in high-baseline performers gamma- (but not alpha) tACS selectively and significantly improved misbinding-feature errors as well as memory precision, particularly in uninformative (invalid) cues which rely more strongly on attentional abilities. We concluded that parietal gamma oscillations, therefore, modulate working memory recall processes, although baseline performance may further influence the effect of stimulation.

## Introduction

Working memory (WM), our ability to hold information in mind for brief periods of time (Baddeley 2012; Gazzaley et al. 2005; Hasher and Zacks 1988; Hasher et al. 2007; Salthouse and Meinz 1995), is intrinsically connected to attentional (Gazzaley and Nobre 2012; Klimesch 2012; Landman et al. 2003; Matsukura et al. 2007; Makovski et al. 2008; Murray et al. 2013), as well as inhibitory processes (Borghini et al. 2018; Klimesch et al. 2007; Sauseng and Klimesch 2008; Waldhauser et al. 2012), which are required to prioritize task-relevant information and suppress task-irrelevant one. Attention and inhibitory-based processes can operate retrospectively on the internally maintained content of working memory by means of retrospective cues (Astle et al. 2012; Matsukura et al. 2007; Myers et al. 2017; Souza et al. 2016). These cues act by protecting information from being removed from the central WM store and in turn by providing a ‘gate’ that filters the information most likely to be relevant for future behavior (Chatham and Badre 2015; Myers et al. 2017; Gazzaley and Nobre 2012).

Some of the abilities relevant for working memory performance have been associated with oscillatory neural activity, especially in the gamma and alpha bands (35–70 Hz and 8–13 Hz, respectively) in the occipito-parietal areas among others (Constantinidis and Klingberg 2016; Gazzaley and Nobre 2012; Herrmann et al. 2013; Jensen et al. 2007; Jensen and Mazaheri 2010; Klimesch et al. 2007; Palva et al. 2010; Rihs et al. 2007; Roux et al. 2012; Tuladhar et al. 2007; Thut 2007; Thut et al 2012). For instance, gamma oscillations are implicated in maintaining the memory of the stimuli to be recalled (Buzsáki and Wang 2012; Howard et al. 2003; Roux and Uhlhaas 2014; Sederberg et al. 2003; Tallon-Baudry et al. 1999), and in re-directing attention to task-relevant stimuli (Buzsáki and Wang 2012; Herrmann et al. 2004; Lachaux et al. 2005; Ray et al. 2008). The contribution of gamma oscillations to this variety of cognitive processes may also be because of their involvement in local cortical activity (Buzsaki 2006; Donner and Siegel 2011; but see Ray and Maunsell 2015).

Another type of attention process that is relevant for working memory performance is in terms of inhibitory abilities, consisting of suppressing task-irrelevant information (Borghini et al. 2018; Klimesch et al. 2007; Sauseng and Klimesch 2008; Waldhauser et al. 2012). These abilities are mainly associated with alpha oscillations (Bonnefond and Jensen 2012, 2013, 2015), as suggested by correlational EEG evidence of successful inhibition of task-irrelevant stimuli in WM tasks when ongoing alpha amplitude is high (Fu et al. 2001; Jensen and Mazaheri 2010; Kelly et al. 2006; Klimesch 1999; Poch et al. 2014; Sauseng et al. 2009; Thut 2007). Alpha power desynchronizes during cognitive task engagement, i.e., it decreases relative to baseline activity in task-relevant brain areas due to less synchronous neural activity (local desynchronization), and as such it suppresses sensory input during parts of the alpha cycle or ‘pulsed inhibition’ (Bonnefond and Jensen 2015; Jensen and Mazaheri 2010; Klimesch et al. 2007). In parallel, during task performance, power increases in gamma oscillations, among other frequency ranges, reflecting active processing of information (Foxe and Snyder 2011; Hanslmayr et al. 2011; Jensen and Mazaheri 2010; Kelly et al. 2006; Sauseng et al. 2009; Thut et al. 2006; Romei et al. 2010; Zanto and Gazzaley 2009). The inhibition of task-irrelevant stimuli and the active processing of task-relevant ones typically corresponds to the coupling between alpha and gamma oscillations (Osipova et al. 2008; Roux et al. 2013). This coupling is such that higher alpha power corresponds to stronger suppression of gamma activity (Bonnefond and Jensen 2015).

While the above studies focused on the correlational role of gamma and alpha oscillations in re-directing attention, in stimuli maintenance as well as in contributing to suppress task-irrelevant information, the modulatory role of these oscillations remains to be established. This can be achieved using transcranial alternating current stimulation (tACS) to experimentally interfere with specific brain oscillations (Antal and Paulus 2013; Battleday et al. 2014; Herrmann et al. 2013; Marshall and Binder 2013; Parkin et al. 2015; Sauseng and Klimesch 2008; Thut et al. 2011), and to assess whether this may in turn modulate the cognitive functions that are thought to rely on these oscillations (Basar et al. 2001; Cecere et al. 2015; Engel et al. 2001; Helfrich et al. 2014a; Herrmann et al. 2004).

Here, we aimed to test the modulatory role of the neural oscillations involved in attention and inhibition in the context of WM. We compared the effects of tACS at gamma and alpha frequency relative to a sham (no stimulation) control condition. We concurrently measured performance in an established WM paradigm (Bays and Husain 2008; Borghini et al. 2018), which required remembering a set of four arrow stimuli varying in color and orientation, and in some trials also entailed re-orienting attention to one of the items held in memory (using retrospective cues). Following a short delay, participants matched the orientation of a probe (one of the arrow stimuli presented in random orientation) to one of the items held in memory with the same color. During the WM maintenance, a retro-cue was presented in 70% of the trials, indicating either the most relevant memory item, in other words the item to be probed later in the trial (valid or informative cue), or hence attention was directed to another item than the would-be probed one via an invalid (or uninformative) cue. In the remaining 30% of the trials, a neutral cue was presented. Retro-cues are known for triggering top-down biasing mechanisms which may either facilitate or impair performance depending on whether the primed item is a target or a non-target (Berryhill et al. 2012; Gazzaley and Nobre 2012; Gozenman et al. 2014; Griffin and Nobre 2003; Landman et al. 2003; Makovski and Jiang 2007; Makovski et al. 2008; Matsukura et al. 2007; Pertzov et al. 2013; Rerko and Oberauer 2013; Tanoue and Berryhill 2012). For each of the retro-cues, this paradigm provides an index of WM accuracy (recall precision), as well as the source of error modulating accuracy, among which are the probability to respond to target orientations and to non-target orientations (Bays and Husain 2008; Bays et al. 2011; Gorgoraptis et al. 2011; Ma et al. 2014; Pertzov et al. 2012, 2013).

Since gamma-tACS may alter gamma power (Helfrich et al. 2014a; Kasten et al. 2016; Neuling et al. 2013; Vossen et al. 2014; Witkowski et al. 2016; but see Antal et al. 2008), we reasoned that a modulatory link between gamma oscillations and stimulus maintenance in WM may result in changes in memory recall regardless of retro-cue type. This modulation is likely to involve the parietal regions because they are known for being part of neural circuits related in both top-down attention and inhibitory processes, which are at the core of our investigation (Constantinidis and Klingberg 2016; Gazzaley and Nobre 2012; Kelly et al. 2006; Klimesch 2012; Tuladhar et al. 2007). Higher recall precision following gamma-tACS may also reflect a modulatory link between of gamma oscillations on top-down attention; this may affect more strongly trials with invalid retro-cues compared to the other cues because these retro-cues require more resources to re-direct attention back to the initial memory array (Astle et al. 2012; Gazzaley and Nobre 2012).

Performance may also be modulated by alpha-tACS, specifically in terms of inhibitory abilities that are relevant to suppress task-irrelevant stimuli (Klimesch et al. 2007; Sauseng and Klimesch 2008; Waldhauser et al. 2012). Changes in inhibitory abilities may be more strongly (although not exclusively) reflected in variations in the probability of target responses (see “Methods” for details), and may be larger or specific to invalidly cued trials. These trials tend to correspond to the largest cost in performance because they require suppressing information that had been invalidly prioritized (see Borghini et al. 2018; Pertzov et al. 2013); hence, by reducing this cost, alpha-tACS may result in the largest changes in invalid trials.

## Materials and methods

### Participants

Fifty-one right-handed stimulation-compatible (Antal and Paulus 2013; Tavakoli and Yun 2017) participants (30 females; age range = 19–34 years, mean = 24.1, SD = 3.6) with normal or corrected-to-normal vision provided written consent to take part in our study that was approved by the local Ethics Committee. None of the participants had past history of neurological or psychiatric disorders, or was under regular medication. Participants received a monetary compensation to complete the experiment.

### Experimental design and task

**Fig. 1:**
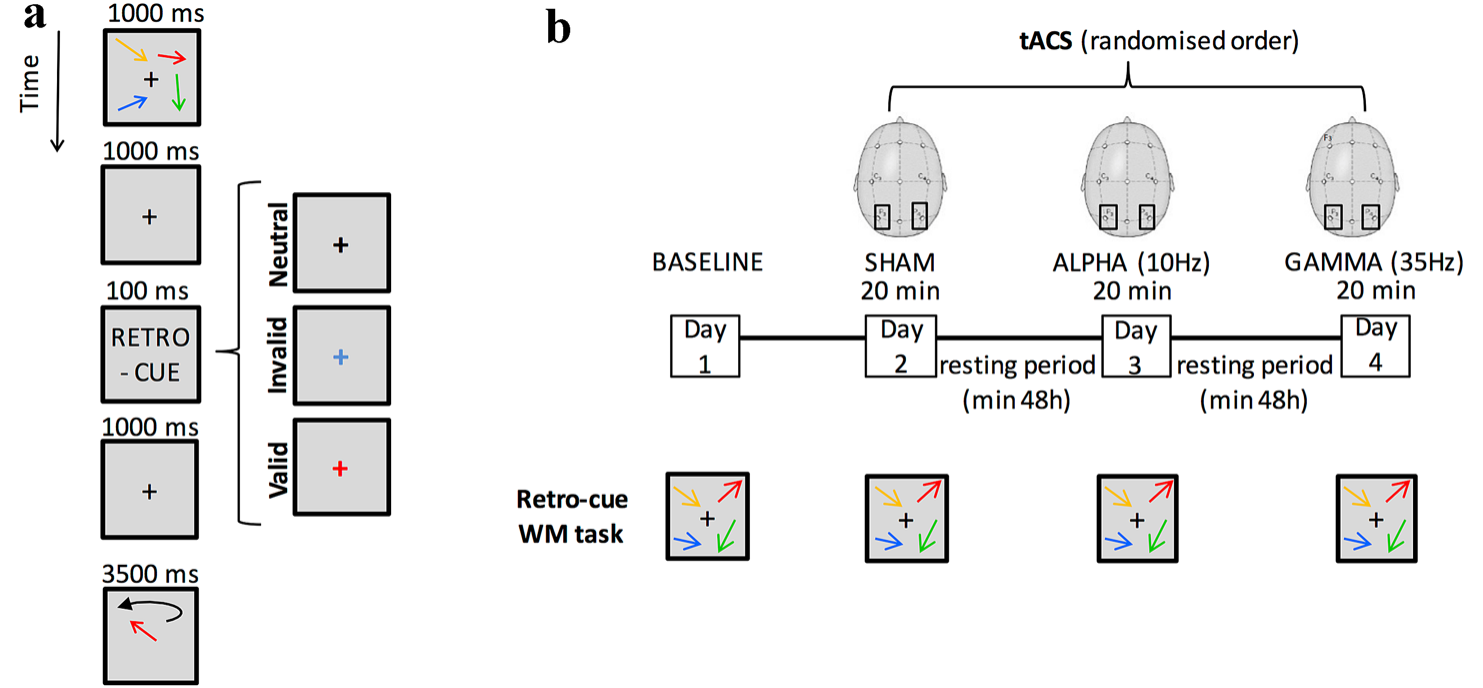
The working memory (WM) retro-cueing task and paradigm. **a** Participants memorized a display of four arrow stimuli differing in orientation and color. Following a delay period, one of the four colored arrows reappeared in a random orientation and participants matched it as closely as possible to the orientation in the original display. In 70% of the trials during the delay, a colored retro-cue was presented highlighting an item that was more likely to be later probed. In these trials, the probe either matched the cued items (validly cued trials, *N* = 62) or it did not (invalidly cued trials, *N* = 26). In the remaining 30% of the trials (*N* = 38), a neutral cue was present during the delay. **b** Participants performed the working memory retro-cue task in a pre-stimulation session with no tACS (baseline), followed by three experimental sessions at least 48 h apart during which they performed the same WM task while receiving 20 min of bilateral parietal (P3 and P4 on 10–20 EEG system) tAC stimulation at either 10 Hz (α band), 35 Hz (γ band), or Sham. The order of the stimulation conditions was pseudo-randomized across participants

Participants used a continuous, analog response to reproduce from memory the feature of a probed item that could be either cued or not cued. If an item was cued, this could be either validly or invalidly (Pertzov et al. 2013; Fig. 1a and experimental stimuli below). The task allowed measuring how precisely a feature of an item (orientation) was recalled, as well as the sources of error accounting for memory performance (see below; Bays and Husain 2008; Ma et al. 2014; Pertzov et al. 2013).

In all experimental sessions, participants sat comfortably in a dimly lit room in front of a 21ʺ CRT monitor with a distance of 60 cm. The stimulation was set to last throughout the task. Matlab 7.0 using the Cogent toolbox (http://www.mathworks.co.uk) was used to program the task, time the stimuli and record the measurement variables of interest (see “Data analysis” below).

### Experimental stimuli

Each trial began with a 500 ms centrally presented black fixation cross (0.8° diameter) displayed on a gray background, followed by a 1000 ms display of four arrows (visual angle: 2° × 0.3°). The arrows were simultaneously presented in four out of five randomly selected and easy distinguishable colors (white, yellow, red, green, blue), pointing in different arbitrary directions with a minimum of 10° difference between the stimuli within a trial. Participants had to keep in mind both the orientation and the color of these arrows.

A total of 126 trials were used, 30% of which (*N* = 38) comprised a neutral cue presented during the memory delay; neutral cues consisted of a white fixation cross that did not change in color. In the other 70% of the trials (*N* = 88), the stimulus display was followed by a 1000 ms delay and by the presentation of an informative retro-cue (for 100 ms). The retro-cue indicated the color of the stimulus arrow most likely to be later probed. Within the 70% trials with an informative retro-cue, 70% (*N* = 62) corresponded to the item that was subsequently probed (valid condition). The remaining 30% of the retro-cue trials (*N* = 26) consisted of items that were subsequently invalidly probed (invalid condition).

All retro-cues were followed by a 3000 ms delay before the presentation of the probe. The probe consisted of a randomly oriented arrow of the same color as one of the arrows in the memory array. Participants adjusted the orientation of the probe to match the remembered orientation with a maximum response time of 3500 ms. In each stimulation condition, participants completed three blocks of the task for a total of 126 trials.

### Stimulation parameters

Participants first performed the WM retro-cue task with no stimulation (baseline). Baseline performance was used to explore individual variability (see “Two-steps cluster analysis” below). Participants subsequently underwent three experimental sessions at least two days apart. In each session, they performed the same WM task while receiving bilateral parietal tAC stimulation at either 10 Hz (α band) or 35 Hz (γ band), or Sham which aimed to exclude any generic learning or fatigue effects (Fig. 1b). The order of the stimulation conditions was counterbalanced and pseudo-randomized across participants.

**Fig. 2:**
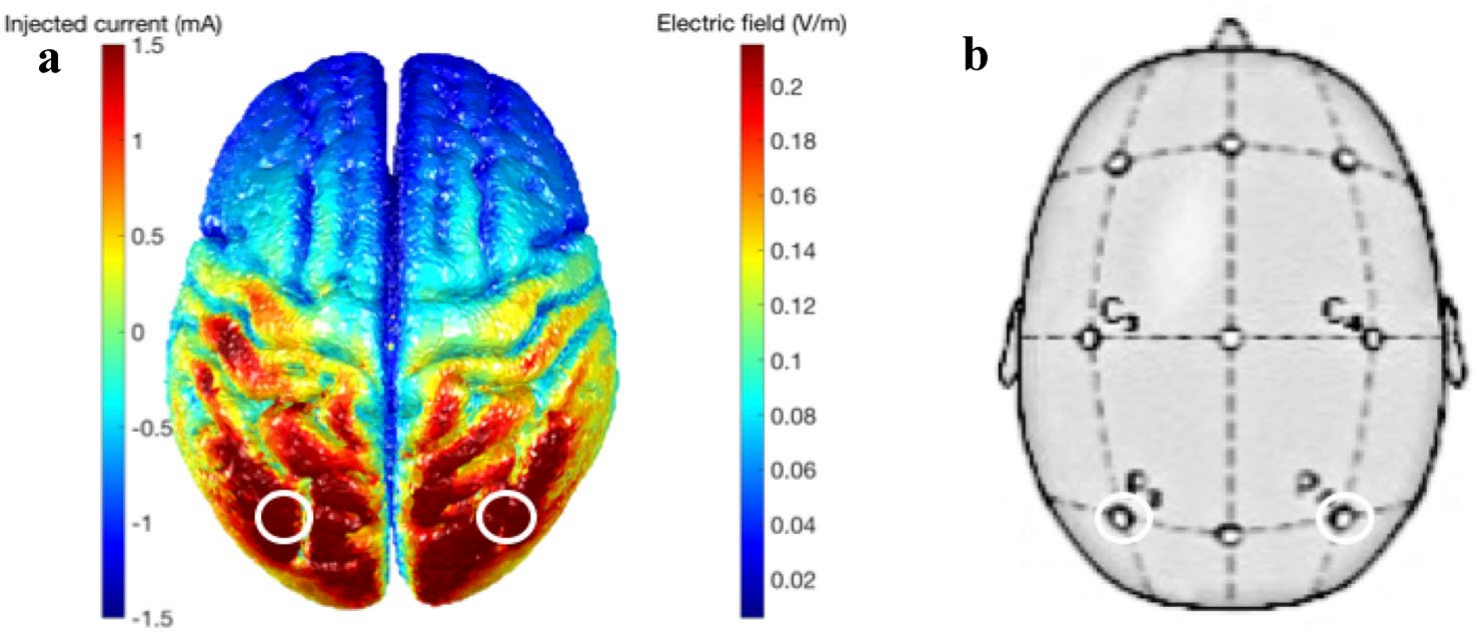
Current modeling. **a** Modeling of the electric field induced by 1.5 mA current of the tAC stimulation to the bilateral parietal lobes, which **b** corresponded to P3 and P4 on the 10–20 EEG system used to locate the target areas. The current modeling performed on ‘ROAST’ (https://www.parralab.org/roast/, Huang et al. 2019) shows a bilateral electric field distribution with maximum current over the posterior parietal areas which we stimulated

A model of the current distribution based on ‘ROAST’ (Huang et al. 2019) shows the bilateral electric field distribution with maximum current corresponding to the stimulated areas in the posterior parietal lobe (Fig. 2).

In all experimental sessions, sinusoidal stimulation was applied with a battery-driven MagStim stimulator and delivered through two 35cm^2^ (5 × 7 cm) conductive rubber electrodes, each covered with a sponge pad soaked in saline solution and positioned over the parietal regions (P3 and P4 following standardised international 10/20 system, Oostenveld and Praamstra 2001). Participants were stimulated at each frequency for 20 min with a strength of 1.5 mA and a fade-in–fade-out period of 20 s, with the exception of Sham. During the Sham condition, the same setting was maintained, but the current was settled at the lowest frequency (4 Hz) and was turned off after 20 s. This allowed successful blinding of the participants to the condition they received, since any initial tingling sensation associated with the sham stimulation was in common with the real stimulation (e.g., Fertonani et al. 2011; Gandiga et al. 2006). At least one of the experimenters was also blind to the experimental condition received by the participants.

### Data analysis

The analysis of performance closely followed that described by Borghini et al. (2018) in their study of older participants.

Recall precision was used as an overall measure of performance, expressed as the reciprocal of the circular standard deviation of error (Fisher 1995). For each trial, the recall error was calculated as the angular deviation between the orientation of the arrow stimulus reported by the participant and the veridical orientation of the target stimulus in the memory array.

Moreover, to investigate the source of participants’ errors in the WM retro-cueing task, we applied an established probabilistic model (see Bays and Husain 2008; Bays et al. 2009, 2011). This assumes that errors in reporting the stimuli orientation arise from three possible sources: the variability in reporting the orientation of the target; mistakenly reporting the orientation of another (non-target) items in the memory array; or just responding at random.

The model is defined by the following equation:

$$p(\hat{\theta }) = a\phi_{\kappa } (\hat{\theta } - \theta ) + \beta \frac{1}{3}\sum\limits_{i}^{m} {\phi_{\kappa } (\hat{\theta } - \varphi_{i} ) + \gamma \frac{1}{2\pi }} ,$$

where *θ* is the true orientation of the target item, ^ the orientation reported by the subject, and *Φ*_*κ*_ is the von Mises distribution (the circular analog of the Gaussian distribution) with mean of zero and concentration parameter *κ*. Concentration parameter *κ* reflects the variability of recall of the target orientation, whereby higher *κ* corresponds to lower variability. The probability of reporting the correct target item (pT) is given by *α*. The probability of misreporting a non-target item (pNT) which can arise when the features of one stimuli are erroneously combined with another, is given by *β*, and {*φ*1, *φ*2,…,*φ*_*m*_} are the orientations of the non-target items. The probability of responding randomly (pU) is given by *γ* = 1−*α*−*β*. Maximum likelihood estimates (Myung et al. 2013) of the parameters *κ*, *α*, *β* and *γ* were obtained separately for each participant, stimulation condition and retro-cue type using an expectation–maximization algorithm.

Performance was analyzed using the generalized estimating equations (GEE) procedure (Zeger and Liang 1986). The GEE is an extension of generalized linear models (GLM) which produces more efficient and unbiased regression estimates for analyzing repeated-measures research designs with non-normal response variables, like in the case of the current data. Performance during stimulation was assessed by fitting repeated-measures regressions, using retro-cue type (valid, invalid and neutral) and stimulation condition (sham, alpha and gamma) as predictors. Within the GEE model, we used the gamma regression with a loglog link function to separately model accuracy (precision) and each index of error (pT, pNT, pU, *κ*). Moreover, the retro-cue mean value of the sham performance for each index was entered as a covariate in each GEE analyses using the ‘robust estimation’ option of the covariance matrix in the model. This aimed to control for differences in performance in the absence of stimulation (here sham) and for the possibility that results may be explained in terms of regression to the mean (Barnett et al. 2005). Significant main effects or interactions were followed by GEE-based planned pairwise comparisons (see Borghini et al. 2018; Santernecchi et al. 2013 for a similar approach).

An inspection of the data indicated a large inter-subject variability in recall precision—taken as an overall measure of performance. This was formally assessed using a multi-level mixed model, with baseline precision entered as dependent variable, retro-cue as fixed factor, and ‘subjects’ as random factor testing for individual variability.

As the mixed model indicated that there were significant individual differences in recall precision (see “Results”) and to obtain an unbiased subdivision of the participants, a two-step clustering procedure was used, with baseline performance across retro-cues as the continuous discriminant variable, following past studies (e.g., López-Alonso et al. 2014). This revealed two almost equal subgroups within the sample, and the subgroup membership (low-baseline and high-baseline performers) was used as additional factor in some post hoc analyses.

Across all performance indexes (accuracy and error) and stimulation conditions in the 51 participants, 33 data points (1.4%) were disregarded because they were deemed outliers as they deviated by more than 3 standard deviations from the group mean. Values reflecting accuracy (precision) and the source of error in all stimulation conditions for the whole sample as well as for low- and high-baseline performers are presented in Table 1.

**Table 1:** Performance during sham and active tACS

Cueing condition	Baseline					Sham					Alpha					Gamma				
	P	*κ*	pT	pNT	pU	P	*κ*	pT	pNT	pU	P	*κ*	pT	pNT	pU	P	*κ*	pT	pNT	pU
Valid																				
All	1.22 *0.03*	2.76 *0.18*	0.82 *0.02*	0.12 *0.02*	0.05 *0.01*	1.30 *0.05*	3.46 *0.4*	0.83 *0.03*	0.11 *0.02*	0.05 *0.02*	1.25 *0.05*	2.9 *0.24*	0.82 *0.03*	0.14 *0.02*	0.03 *0.001*	1.33 *0.0*6	3.01 *0.25*	0.86 *0.02*	0.11 *0.02*	0.05 *0.02*
Low baseline	1.08 *0.03*	2.12 *0.18*	0.79 *0.04*	0.16 *0.03*	0.05 *0.02*	1.08 *0.05*	2.87 *0.7*	0.76 *0.04*	0.17 *0.04*	0.07 *0.03*	1.05 *0.05*	2.12 *0.19*	0.72 *0.04*	0.22 *0.03*	0.05 *0.02*	1.07 *0.04*	2.4 *0.36*	0.77 *0.04*	0.18 *0.03*	0.08 *0.03*
High baseline	1.34 *0.06*	3.34 *0.25*	0.86 *0.02*	0.08 *0.02*	0.06 *0.02*	1.52 *0.07*	4.03 *0.34*	0.90 *0.02*	0.05 *0.02*	0.03 *0.01*	1.46 *0.07*	3.71 *0.38*	0.92 *0.02*	0.07 *0.02*	0.02 *0.005*	1.57 *0.07*	3.6 *0.3*	0.93 *0.02*	0.03 *0.01*	0.03 *0.01*
Invalid																				
All	1.11 *0.04*	3.23 *0.3*	0.69 *0.04*	0.20 *0.03*	0.08 *0.02*	1.17 *0.04*	3.18 *0.26*	0.72 *0.03*	0.20 *0.03*	0.06 *0.02*	1.19 *0.04*	3.3 *0.27*	0.74 *0.03*	0.16 *0.02*	0.08 *0.02*	1.37 *0.08*	3.62 *0.31*	0.75 *0.03*	0.15 *0.02*	0.09 *0.03*
Low baseline	0.99 *0.04*	2.98 *0.44*	0.56 *0.06*	0.31 *0.04*	0.1 *0.03*	1.05 *0.04*	2.56 *0.31*	0.62 *0.06*	0.29 *0.06*	0.06 *0.02*	1.06 *0.05*	2.89 *0.39*	0.67 *0.06*	0.19 *0.03*	0.10 *0.03*	1.03 *0.04*	2.63 *0.37*	0.62 *0.06*	0.22 *0.04*	0.15 *0.05*
High baseline	1.22 *0.06*	3.48 *0.47*	0.80 *0.05*	0.09 *0.03*	0.07 *0.04*	1.30 *0.06*	3.77 *0.37*	0.82 *0.03*	0.12 *0.03*	0.05 *0.02*	1.31 *0.06*	3.71 *0.37*	0.80 *0.03*	0.14 *0.03*	0.06 *0.03*	1.69 *0.13*	4.47 *0.42*	0.87 *0.02*	0.09 *0.02*	0.03 *0.01*
Neutral																				
All	1.11 *0.03*	2.5 *0.18*	0.76 *0.03*	0.19 *0.03*	0.04 *0.02*	1.16 *0.03*	2.73 *0.21*	0.77 *0.03*	0.14 *0.02*	0.08 *0.02*	1.16 *0.04*	2.89 *0.25*	0.77 *0.03*	0.15 *0.02*	0.04 *0.01*	1.25 *0.06*	3.00 *0.26*	0.78 *0.03*	0.17 *0.03*	0.04 *0.01*
Low baseline	1.00 *0.03*	2.3 *0.31*	0.67 *0.05*	0.26 *0.04*	0.05 *0.02*	1.03 *0.03*	2.33 *0.24*	0.66 *0.05*	0.19 *0.04*	0.12 *0.04*	1.02 *0.03*	2.75 *0.38*	0.68 *0.05*	0.21 *0.04*	0.04 *0.02*	1.01 *0.03*	2.35 *0.32*	0.68 *0.05*	0.26 *0.05*	0.06 *0.03*
High baseline	1.21 *0.05*	2.7 *0.20*	0.84 *0.03*	0.12 *0.03*	0.04 *0.02*	1.31 *0.05*	3.1 *0.32 s*	0.88 *0.03*	0.09 *0.02*	0.04 *0.02*	1.31 *0.07*	3.01 *0.33*	0.86 *0.03*	0.1 *0.03*	0.04 *0.02*	1.49 *0.09*	3.61 *0.37*	0.87 *0.03*	0.07 *0.02*	0.03 *0.01*

Mean and standard error (in italics) of accuracy (recall precision) and source of error (*κ*, pT, pNT, pU) in each tACS condition and sham across all participants, as well as separately for high- and low-baseline performers

*P* precision, *κ* kappa, *pT* target responses, *pNT* non-target responses (misbinding errors), *pU* random error

## Results

### Whole sample

All the analyses on the whole sample were based on the generalized estimating equations (GEE) procedure with stimulation and retro-cue type as predictors.

#### Overall performance: recall precision

**Fig. 3:**
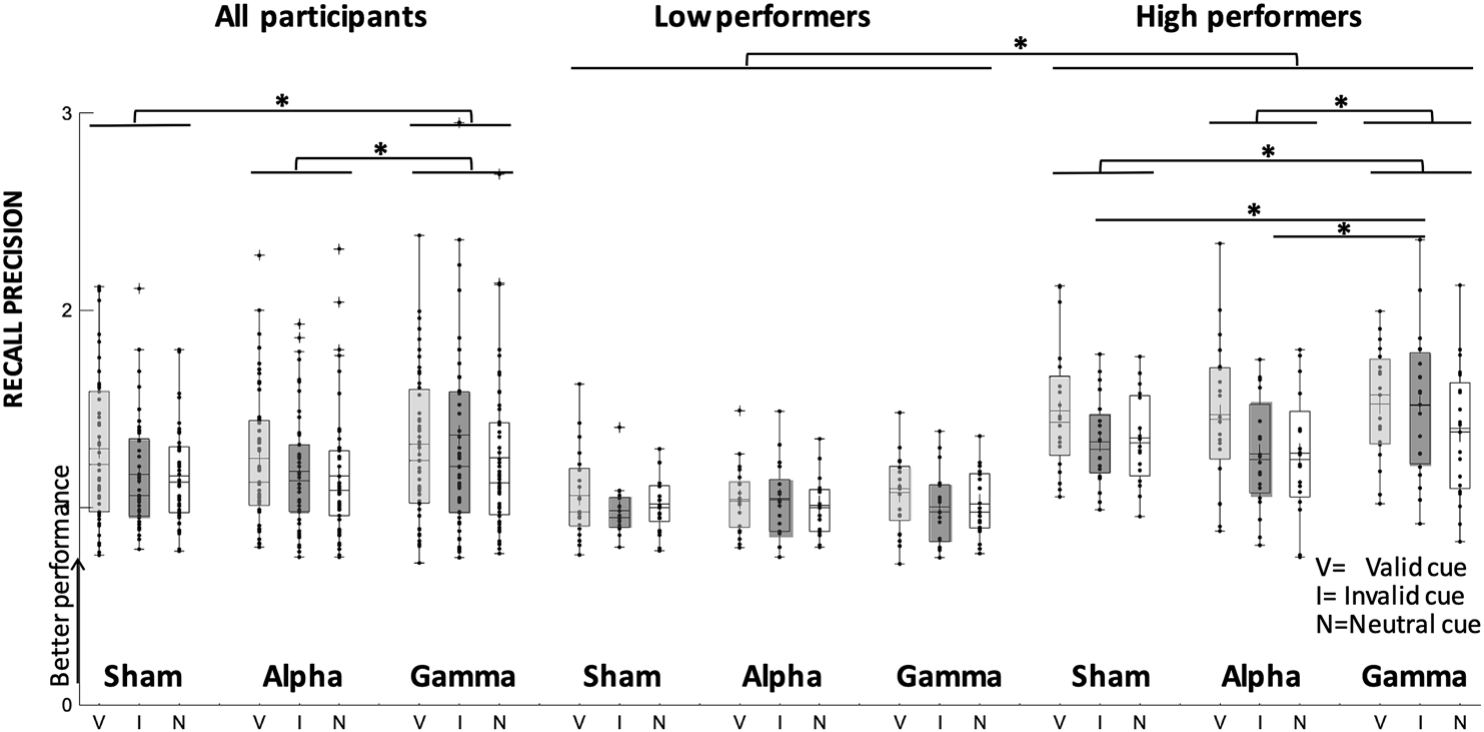
Recall precision during tACS. Changes in recall precision following alpha, gamma-tACS and sham in valid, invalid and neutral retro-cues in the whole sample as well as in low-baseline and high-baseline performers. Each dot indicates a participant’s performance in each condition. Cross symbols refer to the group mean, and asterisks denote significant differences (*p* < 0.05)

Retro-cue type modulated performance across stimulation conditions (main effect of retro-cue, Wald’s *χ*^2^ = 13.4, *p* = 0.001), because recall precision was higher in valid (1.29, SE = 0.05) relative to neutral retro-cues (1.19, SE = 0.04; mean difference = 0.1, *p* = 0.001; Cohen's *d* = 0.5) but not to invalid ones (*p* < 0.07). Regardless of retro-cue type, recall precision was also modulated by the type of stimulation (main effect of stimulation, Wald’s *χ*^2^ = 8.9, *p* = 0.01) because it increased significantly during gamma-tACS (1.32, SE = 0.06) relative to sham (1.21, SE = 0.04; mean difference = 0.1, *p* = 0.02, Cohen's *d* = 0.3) and alpha-tACS (1.2, SE = 0.04; mean difference = 0.11, *p* = 0.005; Cohen's *d* = 0.3), see Fig. 3. No other effects reached significance (Table 1).

#### Probability to respond to the target orientation

**Fig. 4:**
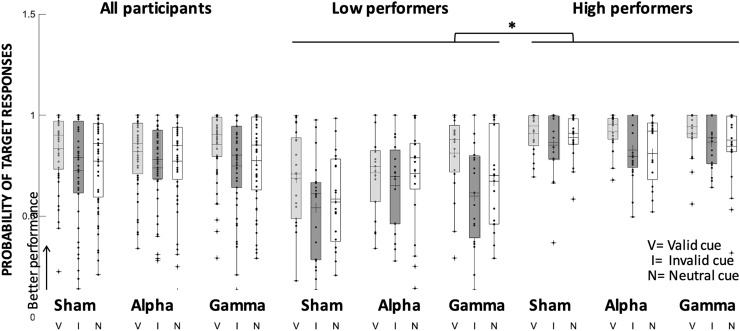
Probability of target responses (pT) during tACS. Changes in the probability of target responses following alpha, gamma-tACS and Sham in valid, invalid and neutral retro-cues in the whole sample as well as in low-baseline and high-baseline performers. Each dot indicates a participant’s performance in each condition. Cross symbols refer to the group mean, and asterisks denote significant differences (*p* < 0.05)

Retro-cue type modulated target responses across stimulation conditions (main effect of retro-cue, Wald’s *χ*^2^ = 18.3, *p* < 0.001). Performance was again better in valid trials (0.84, SE = 0.02) relative to invalid (0.74, SE = 0.03; mean difference = 0.09, *p* = 0.001; Cohen's *d* = 0.2) and to neutral ones (0.77, SE = 0.03; mean difference = 0.06, *p* = 0.002; Cohen's *d* = 0.2), see Fig. 4 and Table 1. No other effects reached significance.

#### Misbinding: probability to respond to non-target orientations

**Fig. 5:**
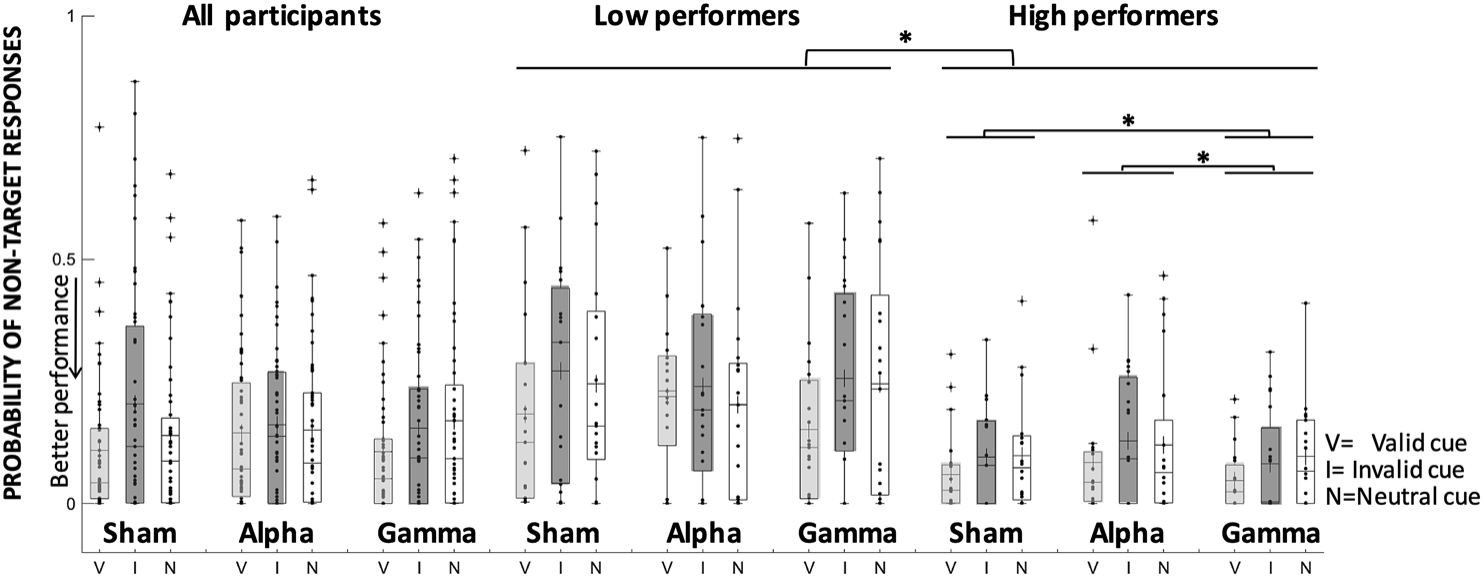
Probability of non-target responses (pNT) during tACS. Changes in the probability of non- target responses following alpha, gamma-tACS and Sham in valid, invalid and neutral retro-cues in the whole sample as well as in low-baseline and high-baseline performers. Each dot indicates a participant’s performance in each condition. Cross symbols refer to the group mean, and asterisks denote significant differences (*p* < 0.05)

Retro-cue type modulated misbinding errors across stimulation conditions (main effect of retro-cue, Wald’s *χ*^2^ = 5.8, *p* = 0.05), since participants still made significantly fewer misbinding errors in valid trials (0.12, SE = 0.02) relative to invalid and neutral ones (respectively, 0.17, SE = 0.02 and 0.15, SE = 0.02; mean difference = − 0.05, *p* < 0.02 and − 0.03, *p* < 0.05, Cohen's *d* = 0.28 and d = 0.2), see Fig. 5 and Table 1. No other effects reached significance.

#### Random error

Retro-cue type modulated random responses across stimulation conditions (main effect of retro-cue, Wald’s *χ*^2^ = 7.8, *p* = 0.02). There were significantly fewer random errors in valid retro-cues (0.04, SE = 0.008) relative to invalid (0.07, SE = 0.02; mean difference = − 0.03, *p* = 0.009; Cohen's *d* = 0.25) but not neutral (0.05, SE = 0.009; mean difference = − 0.008, *p* = 0.4), see Table 1. No other effects reached significance.

#### Concentration parameter (*κ*)

No significant effects of retro-cue or stimulation, or their interaction were detected (Table 1).

### Low- vs high-baseline performers

An inspection of the precision data indicated large individual variability in performance, supported by the results of a mixed-model analysis showing a significant random intercept (Wald’s *Z* = 8.5, *p* < 0.001). To take into account this individual variability, and to identify possible independent subgroups while obtaining a blind splitting of the participants, we run a two-cluster analysis with the average baseline performance across retro-cues as continuous variable, the number of clusters automatically determined and evaluated with the Bayesian information criterion (BIC) goodness-of-fit statistic. This resulted in two almost equal subgroups consisting of about 51 and 49% of the total sample (26 and 25 participants, respectively), whose recall precision across retro-cues differed significantly (low-baseline performers: 1.02, SE = 0.08; high-baseline performers: 1.26, SE = 0.08; *Z* = − 4.6, *p* < 0.001, Cohen’s *d* = 0.56). In line with previous data in young adults (Borghini et al. 2018; Pertzov et al. 2013), recall precision at baseline benefitted from valid retro-cues relative to neutral ones in both groups (low-baseline performers: *Z* = 2.8, *p* = 0.005, Cohen’s *d* = 0.4; high-baseline performers: *Z* = 2.2, *p* < 0.03, Cohen’s *d* = 0.4) but it was not significantly weakened by invalid retro-cues.

To test whether group variability had an effect on performance, we, therefore, performed a second round of GEE analyses for each index of performance, with subgroup, stimulation and retro-cue as predictors and the retro-cue mean value of sham performance as a covariate to control for pre-stimulation performance.

#### Overall performance: recall precision

Across retro-cues, the effect of stimulation on recall precision differed in the two subgroups (interaction of stimulation and subgroup: Wald’s *χ*^2^ = 9.5, *p* < 0.009), because in high-baseline performers it increased significantly following gamma-tACS relative to sham (mean difference = 0.20, *p* = 0.013, Cohen's *d* = 0.43) and to alpha-tACS (mean difference = 0.22, *p* = 0.002; Cohen's *d* = 0.46) (Table 1; Fig. 3). In contrast, recall precision in low-baseline performers did not significantly change following gamma- and alpha-tACS relative to sham (all *p*s > 0.4).

The impact of stimulation in the two subgroups also depended on the type of retro-cue (significant triple interaction, Wald’s *χ*^2^ = 9.5, *p* < 0.05). Subsequent GEE analyses independent for each subgroup indicated that this interaction was driven by a significant change in recall precision in high-baseline performers, modulated by retro-cue type and stimulation (significant interaction, Wald’s *χ*^2^ = 10.4, *p* = 0.03). Specifically, participants in this group showed higher recall precision in invalid retro-cues following gamma-tACS relative to sham (mean difference = 0.38, *p* = 0.007; Cohen's *d* = 0.75) and to alpha-tACS (mean difference = 0.37, *p* = 0.009; Cohen's *d* = 0.66), and relative to low-baseline performers in the same stimulation and retro-cue type (mean difference between high- and low-baseline performers = 0.65, *p* < 0.001; Cohen's *d* = 1.2). No effects reached significance in low-baseline performers (Table 1; Fig. 3).

#### Probability to respond to the target orientation

Across stimulation conditions and retro-cue types, target responses differed in low- and high-baseline performers (main effect of subgroup, Wald’s *χ*^2^ = 25.3, *p* < 0.001), because they were significantly higher in high-compared to low-baseline performers (0.87, SE: 0.01 vs 0.69, SE: 0.03, mean difference = 0.18, *p* < 0.001; Cohen's *d* = 2.5). Target responses in the two subgroups also differed significantly as a function of retro-cue and stimulation (significant triple interaction, Wald’s *χ*^2^ = 10.4, *p* < 0.04). However, subsequent GEE analyses for each subgroup showed that no effects reached significance (Table 1; Fig. 4).

#### Misbinding: probability to respond to non-target orientations

Non-target responses across stimulation conditions and retro-cue type differed significantly in the two subgroups (main effect of subgroup, Wald’s *χ*^2^ = 52.5, *p* < 0.001), because they were significantly higher (worse performance) in low- than high-baseline performers (0.19, SE: 0.02 vs 0.08, SE: 0.007).

Regardless of retro-cue type, group differences in the proportion of misbinding errors continued to be modulated by stimulation (interaction of subgroup and stimulation, Wald’s *χ*^2^ = 6.1, *p* < 0.05). This is because misbinding errors decreased significantly in high-baseline performers, specifically following gamma-tACS relative to sham (mean difference = − 0.03, *p* < 0.05; Cohen's *d* = 0.51) and alpha (mean difference = − 0.46, *p* < 0.01; Cohen's *d* = 1), and they did not change in low-baseline performers (all *p*s > 0.7), see Table 1, Fig. 5. No other effects reached significance.

#### Random error

Low-baseline performers made significantly more random errors than high-baseline performers regardless of stimulation and retro-cues (0.06, SE: 0.01 vs 0.04, SE: 0.004; main effect of subgroup Wald’s *χ*^2^ = 12.7, *p* < 0.001). No other effects reached significance (Wald’s *Z* = 0.13, *p* = 0.8).

#### Concentration parameter (*κ*)

Regardless of stimulation and retro-cue type, random responses of high-baseline performers showed significantly larger variability than low-baseline performers (2.6, SE: 0.2 vs 3.6, SE: 0.3; main effect of subgroup, Wald’s *χ*^2^ = 18.2, *p* < 0.001). No other effects reached significance.

## Discussion

Using brain stimulation coupled with a WM retro-cueing paradigm, we investigated whether gamma and alpha oscillations may modulate stimuli maintenance, top-down attention, and inhibitory abilities, namely the capacity to ignore task-irrelevant information. These cognitive processes have often been correlationally associated with gamma and alpha oscillations (Bonnefond and Jensen 2013,2015; Bastos et al. 2012; Bauer et al. 2014; Gazzaley and Nobre 2012; Klimesch 2012; Klimesch et al. 2007; Landman et al. 2003; Matsukura et al. 2007; Makovski et al. 2008; Murray et al. 2013; Sauseng and Klimesch 2008; Waldhauser et al. 2012), hence our choice to experiment their possible modulatory role.

Our results revealed three main findings. First, recall precision improved significantly following parietal gamma stimulation relative to alpha and sham stimulation. Second, there was significant and large individual variability in performance before stimulation (baseline) as well as during stimulation. Only about half of the participants (high-baseline performers) showed significantly better recall precision, higher probability of target responses, lower misbinding errors (non-target responses), and larger variability in their responses. Third, only in high-baseline performers, parietal gamma-tAC stimulation led to a significant improvement in recall precision, which was higher in the case of invalid cues. This enhancement was accompanied by a significant gamma-tACS-based decrease in misbinding errors (better performance) across retro-cues.

Two possible, not mutually exclusive explanations may account for the increased recall precision following parietal gamma stimulation that we observed. Gamma-tACS may have facilitated maintaining the memory of the initially presented stimuli, or it may have facilitated re-directing attention. Gamma oscillations are thought to reflect active processing of information (Bertrand and Tallon 2000; Fries 2005), which may facilitate maintaining stimuli in working memory. For instance, EEG evidence suggests that gamma power is higher during the maintenance in WM of the stimuli to be remembered (Howard et al. 2003; Palva et al. 2010; Roux et al. 2012; Tallon-Baudry et al. 1999). In the current study, although EEG data are needed to corroborate our findings, gamma-tACS may have contributed to increase gamma power, as shown in other tACS studies (Helfrich et al. 2014a; Kasten et al. 2016; Neuling et al. 2013; Vossen et al. 2014; Witkowski et al. 2016), which in turn resulted in higher recall precision in all retro-cues.

In some of our participants and specifically in high-baseline performers, improved recall precision following gamma-tACS was even higher in the case of invalid retro-cues. This may be because these retro-cues typically require ignoring the stimuli incorrectly prioritized and re-directing attention to the target stimuli in the original display. These processes usually result in a decrease in precision relative to valid cues, as shown in our sham condition and also consistent with previous data (Bays and Husain 2008; Borghini et al. 2018; Ma et al. 2014; Pertzov et al. 2013). The cost of performing invalid cues was reduced following stimulation in the gamma frequency, which is known for being associated to re-orienting of attention (Bauer et al. 2012; Engel et al. 2001). This suggests that besides improving maintenance, gamma-tACS may have contributed to modulating processes related to re-directing attention that are more strongly required to manage invalid cues. We note, however, that this explanation may require further corroboration as in our sample baseline recall precision in invalid retro-cues differed significantly from valid, but not from neutral retro-cues.

These explanations point to potentially distinct roles of gamma oscillations, although the apparent functional relevance of these oscillations across a variety of cognitive functions, including memory and attention, may be because gamma reflects local excitatory–inhibitory cortical interactions which may support the communication between cortical areas and in turn support a number of cognitive processes (Buzsaki 2006; Donner and Siegel 2011; Koppel et al. 2000; but see Ray and Maunsell 2015).

Different from our expectations and from a previous study in aging adults (Borghini et al. 2018), we found no significant changes in performance following alpha-tACS. There may be different factors accounting for this, for instance variability in endogenous alpha oscillations or in cortical excitability in younger adults, two indexes that future studies may focus on to fully understand the effects of tAC stimulation. Since there was no evidence of significant changes related to alpha-tACS, our data do not support the alpha-gamma coupling (Osipova et al. 2008; Roux et al. 2013). Therefore, higher alpha power did not correspond to behavioral changes reflecting stronger suppression of gamma activity, although tACS-based changes in oscillations may have been detectable by electrophysiological measures (Bonnefond and Jensen 2015). We also note that other brain oscillations may play a role in working memory processes, for instance working memory performance is known to be associated with theta power enhancement typically over frontal regions (Hsieh and Ranganath 2014; Kahana et al. 2001; Klimesch 1999; Mitchell et al. 2008). Theta oscillations reflect more strongly the maintenance of items presented sequentially and with progressively increasing load (e.g., Hsieh et al. 2011; Jensen 2006; Lisman and Jensen 2013; Meltzer et al. 2007, 2008; Roberts et al. 2013; Scheeringa et al. 2009). Since these two factors were not manipulated in our design, the role of theta oscillations may be examined in future studies.

A number of post hoc analyses indicated that stimulation effects differed remarkably across our participants, such that in low-baseline performers alpha-tACS modulated responses but not differently from gamma-tACS, which itself did not differ from Sham. Lack of unambiguous stimulation effect in these participants is unlikely to be related to their poorer recall precision at baseline, which was overall comparable (i.e., within 1 SD) to that reported in previous studies that included younger adults (e.g., Borghini et al. 2018). Poorer performance was, therefore, unlikely to be due to participants not following the task’s instructions, a reason that does not explain the unclear stimulation effects. In contrast, in high-baseline performers, gamma-tACS resulted in a significant decrease of misbinding errors, and higher recall precision. Therefore, high- rather than low-baseline performance corresponded to larger gamma-tACS benefits, in agreement with some (e.g., Jones and Berryhill 2012) but not all previous brain stimulation studies (Heinen et al. 2016; Santernecchi et al. 2013), and in line with evidence from other fields such as education (e.g., Duncan et al. 2007). This larger effect of parietal gamma-tACS in high-baseline performers may be due to differences in baseline performance, although this is not always a critical element (e.g., Learmonth et al. 2015), or to other factors, for instance differences in biological substrates such as neurotransmitters distribution, cortical excitability and inhibition or their balance, brain structure or function (Krause et al. 2013; Krause and Cohen 2014). It is also possible that in high-performers, neurons excited by the stimulation become even more active, and inhibited neurons become even less active—a pattern also referred to as ‘rich got richer’ (Donner and Nieuwenhuis 2013). Participants in the two groups may have also differed in terms of their ability to flexibly manipulate their memory representations, a skill typically underlying the effective use of retro-cue (Nobre et al. 2004; Zanto and Gazzaley 2009). Future study may examine resting-state EEG in the context of WM to test whether being able to flexibly manipulate the information carried by retro-cues corresponds to the flexibility in the neural dynamics during resting state.

tACS has been implemented to either entrain (Ozen et al. 2010; Zaehle et al. 2010; but see Vossen et al. 2014) or desynchronize oscillatory activity (Strüber et al. 2014; Guerra et al. 2016). We suggest that improved recall precision following stimulation may be due to gamma-tACS amplifying neuronal activity in the frontoparietal network based on the phenomenon of resonance (Buzsaki 2006). Resonance entails that matching the endogenous oscillation of brain networks supporting a particular cognitive task with the frequency of tAC stimulation may result in augmenting the activity of these networks and their coherence, i.e., neuronal synchronization (Herrmann et al. 2013). This is because tACS is thought to promote a wider recruitment of neurons specific for a cognitive function into rhythmically firing networks (Herrmann et al. 2013; Battleday et al. 2014), which in turns is likely to result in behavioral changes in activities subserved by these neurons. Specifically, increased neuronal activity via gamma-tACS may have amplified the processing of incoming information typically associated with gamma oscillations (Bertrand and Tallon 2000; Bonnefond and Jensen 2013, 2015; Fries 2005). In turn, gamma-tACS increased the processing of incoming information, therefore reinforcing recall precision.

tACS-driven synchronization of oscillatory activity may have been induced by our electrode montage targeting homologous areas in the two hemispheres, similar to previous studies (Helfrich et al. 2014b, c; Strüber et al. 2014; Saturnino et al. 2017). Synchronizing endogenous oscillations in distinct brain areas may occur with an in-phase stimulation (Neuling et al. 2013; Polanía et al. 2012; Tseng et al. 2016), and it is typically linked to improved cognitive performance because synchronized oscillatory brain activity promotes information transfer within functional brain networks by means of long-range neuronal coupling (Buzsaki and Draguhn 2004; Canolty and Knight 2010).

We note, however, that tACS effects may depend on a number of factors such as the task relevance, the distance between the stimulated areas, the cognitive task used or the pre-stimulated brain or cognitive state (Krause and Cohen 2014). This may explain why low-baseline performers showed more generic tACS-driven effects, suggesting that these participants were susceptible to stimulation, although the generality of the effects does not grant further explanations.

In conclusion, we found that parietal gamma-tACS can modulate WM recall precision, especially in high-baseline performers. This improvement—possibly supported by the boosting of stimulus maintenance or selective attention mechanisms—highlights the modulation of parietal gamma oscillations on working memory processes, and shows that baseline performance may influence the effect of stimulation.

## Data Availability

Data and codes are available upon request.
